# Serial percutaneous endoscopic necrosectomy (SPEN) after initial VARD for necrotizing pancreatitis: a retrospective single-center observational study

**DOI:** 10.1007/s00464-026-12587-x

**Published:** 2026-02-04

**Authors:** Julian Palzer, Till Herbold, Karim Hamesch, Marcel Binnebösel, Henning Zimmermann, Georg Wiltberger, Alexander Koch, Florian Vondran, Anjali A. Roeth

**Affiliations:** 1 Department of General, Visceral, Pediatric and Transplant Surgery, RWTH University Hospital Aachen, Pauwelsstr. 30, 52074 Aachen, Germany; 2 Department of Gastroenterology, RWTH University Hospital Aachen, Aachen, Germany; 3 Interdisciplinary Department of Endoscopy, RWTH University Hospital Aachen, Aachen, Germany; 4 Department of General and Visceral Surgery, Klinikum Bielefeld, Bielefeld, Germany; 5 Department of Gastroenterology, St. Antonius Krankenhaus, Eschweiler, Germany

**Keywords:** Acute pancreatitis, Necrotizing pancreatitis, Endoscopic necrosectomy, VARD, Step-up approach, WON, Walled-off-necrosis, SPEN

## Abstract

**Introduction:**

Necrotizing pancreatitis (NP), a severe form of acute pancreatitis (AP), is linked to lower survival rates. Treatment strategies have shifted towards less invasive, step-up approaches, favoring minimally invasive procedures. In this study, we report on the potential of combining the minimally invasive surgical approach with endoscopic necrosectomy as a novel treatment strategy, termed serial percutaneous endoscopic necrosectomy (SPEN), for patients with therapy-refractory NP.

**Material and Methods:**

A cohort of 19 patients suffering from therapy-refractory NP, defined as persistent necroses after drainage treatment and subsequent video-assisted retroperitoneal debridement (VARD), was treated with SPEN upon failure of the above stated. In contrast to surgery, SPEN does not require general anesthesia or an operating theater. The results were compared with the current data on alternative treatments.

**Results:**

The investigated cohort consisted of severely ill patients as most patients experienced organ failure as well as severe disease progression in need of intensive-care unit admission. SPEN was performed 4.3 ± 3.8 times, ranging from 1 to 14 procedures per individual. According to the current Clavien–Dindo classification, only “mild” and no major SPEN procedure-associated complications can be observed.

**Conclusion:**

In this report, we present our experience with a novel treatment approach combining surgery and endoscopic interventions for the treatment of NP. While sparing resources, SPEN was shown to be safe and effective. Favorable implications are implied owing to the combination of the best of two worlds: surgery, with its capacity for extensive necrosectomy and endoscopic necrosectomy, which is valued for its applicability as a flexible, low-grade invasive but effective tool that may be dynamically employed depending on individual disease progression.

Acute pancreatitis (AP) is a common disease with its highest disease burden in regions with high socio-economic status such as Germany or the USA [1] and a globally increasing incidence [2]. This increase in incidence may be attributed to the forecoming of unhealthy lifestyle habits such as excessive alcohol consumption [1] or biliary etiology [2]. In Germany, incidence of acute pancreatitis ranges from 13 to 43 cases per 100.000 citizen per year [3], resulting in 50.000 hospital admissions per year [4].

Among other severe complications, one complication defining severe pancreatitis is the development of peripancreatic necrosis, the so-called necrotizing pancreatitis, which occurs in 5–10% of all patients [5]. Often these necroses develop a surrounding capsule, then called walled-off necrosis (WON). The occurring necroses are primarily sterile. However, in 33% of patients with necrotizing pancreatitis, such necroses become infected. Due to such superinfection or further complications such as vascular erosion, necrotizing pancreatitis is associated with a particularly unfavorable prognosis in terms of increased morbidity and mortality [3, 4].

Due to elaborate efforts to predict the outcome of patients with acute pancreatitis, numerous scores have been established. The most used scoring systems for classifying acute pancreatitis severity is the Revised Atlanta classification (RAC). Exemplarily, the RAC system defines three stages: “mild,” “moderate,” and “severe.” These categories correspond to varying mortality rates, ranging from 0.1% in mild cases to 39.2% in severe cases [6], particularly related to superinfection of necrosis. The widely accepted crucial determinant for mortality in acute pancreatitis is the presence or absence of persistent organ failure. Absence of persistent organ failure eventuates in a mortality of 6–11% while persistent organ failure is associated with a markedly increase of mortality of 36–50% [5].

Yet, despite the global increase in incidence of acute pancreatitis, its mortality has been steadily declining over the course of the last few decades [1]. This may be attributed to advances in treatment of acute pancreatitis in a multidisciplinary, step-up approach.

Early stages of acute “mild” or “moderate” acute pancreatitis are treated conservatively, while the occurrence of infection in necrotizing pancreatitis results in a shift in treatment strategies from a conservative treatment approach to a minimally invasive treatment approach.

The treatment of necrotizing pancreatitis has dramatically changed over the course of the last few decades. The findings of the 2010 PANTHER (PAncreatitis, Necrosectomy versus sTEp up appRoach) trial sparked a trend towards minimally invasive therapy regimes starting with the replacement of the open-necrosectomy with a less invasive a step-up approach [7]. This step-up approach included an initial CT-guided drainage to evacuate the liquid parts of the peripancreatic necrosis. Larger or solid necrosis formations require additional treatment. In adherence with the step-up approach, this was achieved by surgery in terms of a minimally invasive video-assisted retroperitoneal debridement (VARD)[7]. The PANTHER trial proved superiority of this step-up approach over open-necrosectomy in terms of a significant reduction in the occurrence of secondary complications such as organ failure, incisional hernia, new-onset diabetes, use of pancreatic enzymes as well as new-ICU administrations [7].

The 2018 TENSION Trial of the Dutch Pancreatitis Study group compared this surgical minimally invasive step-up approach with an endoscopic minimally invasive step-up approach and demonstrated therapeutic equivalence for both therapies. Aside fewer pancreatic fistula rates as well as lower re-interventions rates in the endoscopic group, both groups did not differ regarding the primary end points of the study such as death or major complications [8, 9].

Within this step-up approach which now represents the gold-standard, the peripancreatic necrosis is primarily addressed with a CT-guided percutaneous drainage or if suitable an endosonographic-ultrasound-guided (EUS-guided) transgastric drainage [10, 11]. The latter may be achieved by implantation of a transgastric stent with or without an additional bridging stent [12]. Yet, drainage-based treatment presents with two major limitations as solid necrosis formations often remain unfazed and progressive necrosis formation may exceed the drainage capacity of drainages, therefore, impeding therapy success.

Consequently, in cases of persistent therapy-refractory peripancreatic necrosis development despite drainage treatment additional treatment is needed. Video-assisted retroperitoneal debridement (VARD) is widely accepted as the next therapy escalation [10]. VARD procedure refers to the surgical necrosectomy using a laparoscopic instruments alongside an existing percutaneous retroperitoneal drainage.

The concept of minimally invasive necrosectomy was further explored in numerous studies. Latest investigations show the potential of percutaneous endoscopic necrosectomy (PEN) [13, 14], which refers to a necrosectomy through a metal stent which is placed percutaneously along an existing percutaneous drainage. Likewise, other minimally invasive percutaneous techniques with a retroperitoneal approach such as the so-called MARPN (Minimal access retroperitoneal pancreatic necrosectomy) procedure have demonstrated beneficial characteristics. MARPN is a percutaneous piecemeal necrosectomy through a canal provided by prior drainage implantation using a nephroscope forceps showed lower mortality and complications rates when compared to open-necrosectomy[15].

In this study, we propose an alternative novel step-up approach combining VARD and serial percutaneous endoscopic necrosectomy (SPEN) for selected patients with therapy-refractory disease. This retrospective observational study aimed to investigate the potential of SPEN as a novel treatment approach for patients with therapy-refractory acute necrotizing pancreatitis.

## Materials and methods

**Fig. 1:**
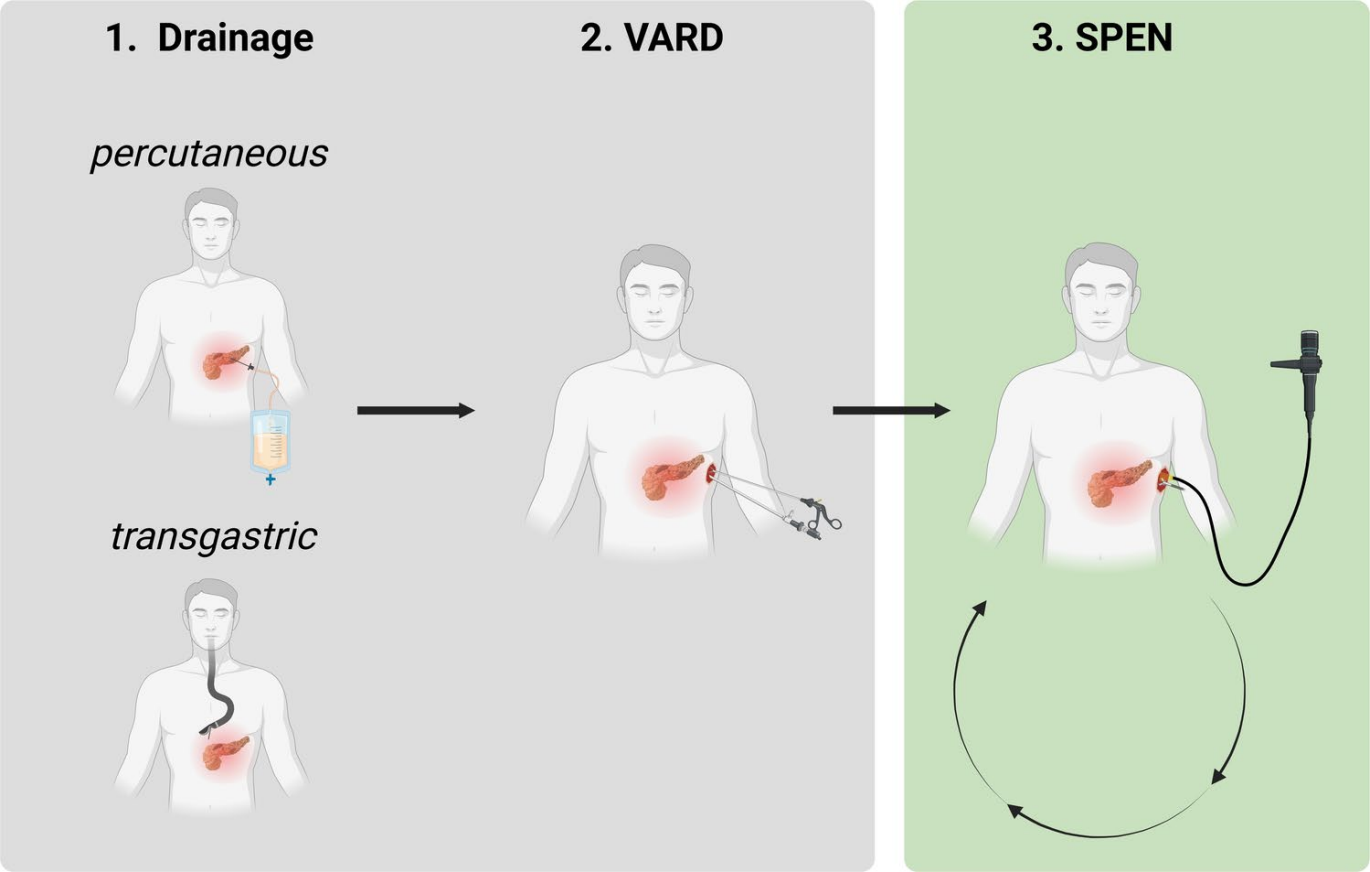
Step-up approach for SPEN

Patients included in this retrospective study had therapy-refractory necrotizing pancreatitis treated between 2016 and 2023 at the Aachen University Hospital, Germany. Therapy-refractory disease was defined as failure to gain sufficient disease control with the conventional step-up approach in terms of drainage and subsequent surgical VARD treatment. A schematic demonstration of the therapy algorithm applied is demonstrated in Fig. 1. In detail, patients initially received a drainage treatment in accordance to the current state of art and adhered to principles described in the TENSION trial [8, 10].

Initially, patients received drainage treatment through EUS-guided transgastric or CT-guided percutaneous drainage. Due to incompatible anatomical circumstances in terms of distant necrosis related to the gastric wall not all patients were eligible for EUS-guided transgastric drainage. Hence, the majority received primary CT-guided percutaneous drainage. Yet, a minority of patients were primarily treated with EUS-guided transgastric drainage. Due to insufficient disease control despite EUS-guided transgastric drainage, these patients flowingly received a CT-guided percutaneous drainage.

**Fig. 2:**
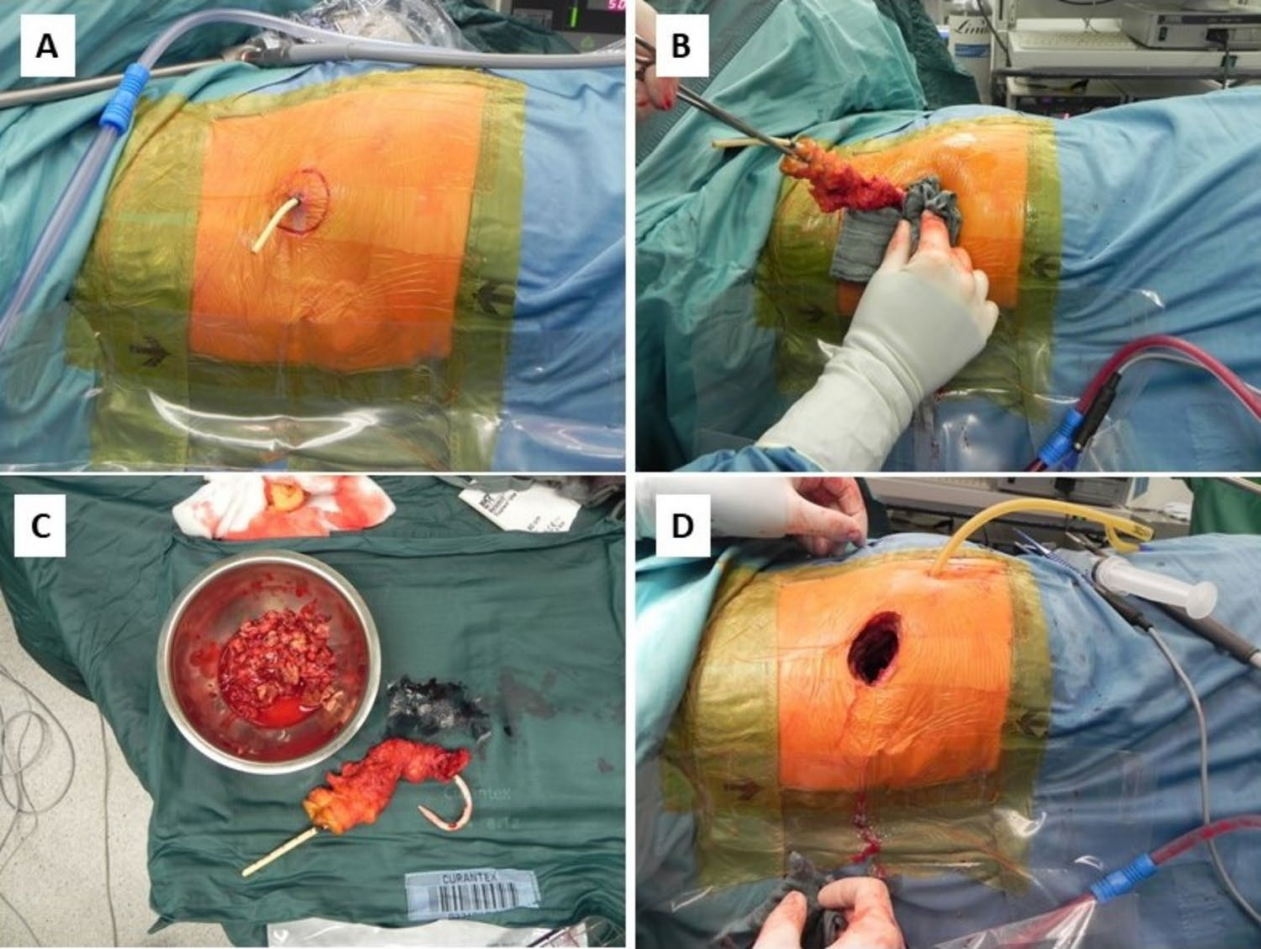
VARD procedure

Upon failure of drainage-based treatment, VARD was employed along the percutaneous drainage channel in order to mobilize large amounts of existing necrosis. VARD was performed in an operating room under general anesthetics, using a laparoscope to ensure visual guidance during the channel preparation. A typical VARD procedure is depicted in Fig. 2 with exemplary intraoperative images. Once general anesthesia and patient positioning were completed, circular skin incision is performed around the preexisting CT-guided drainage before preparation along the drainage using a laparoscope. Preparation finds an end once the necrotic caverns are reached, and the necrotic areas can be evacuated adequately. Hence, a channel for later endoscopic necrosectomy is created. Given persistent necrosis development after VARD, additional SPEN was performed through the existing VARD channel until adequate disease control had been achieved and the need for interventions had ceased, particularly when one or more of the following criteria were met: (1) persistent ICU dependency and/or 2) ongoing systemic infection despite anti-infective therapy (fever and/or laboratory signs of infection). The timing of SPEN repetition was adjusted according to the following criteria: (1) dynamic evaluation of laboratory inflammatory markers (in case of persistent or inclining inflammatory markers alternative sources of infection such as pneumonia or urosepsis had to be ruled out, (2) imaging findings such as persistent or progressive necrosis formations, (3) clinical appearance in terms of signs of systemic infection or sepsis (again alternative infectious foci had to be ruled out). SPEN was performed in supine position using a standard gastroscope as well as regular endoscopic instruments such as endoscopic forceps, scissors, loops, or dormia baskets. As the abdominal cavity was not entered during VARD, the retroperitoneal channel usually remained patent due to the absence of intra-abdominal pressure, hence, allowing for standard necrosectomy without CO2 insufflation. In rare cases, however, gravitational forces caused partial cannel collapse, in these situations, continuous CO2 insufflation was used to maintain patency and adequate visualization. Routinely a silicon-coated latex or 100% silicone 16-French (Fr) catheter was placed inside the VARD channel. This catheter served two main purposes: (1) it enabled irrigation if the VARD channel at any stage of the treatment to allow for drainage of liquid necrosis part in between interventional treatments, and (2) it helped maintain patency of the VARD channel during later stages, when the infection had largely resolved and granulation tissue formation began. Prevention of VARD channel occlusion at these later stages was indicated to avoid secondary infection resulting from entrapment of residual infected tissue after closure of the superficial channel entrance.

In contrast to VARD, endoscopic necrosectomy was conducted under analgosedation (propofol monotherapy or propofol in combination with another agent, e.g., benzodiazepine) administered either by a trained nurse, the endoscopist or an intensivist, in case the patients were bound to the ICU at the time of the intervention. Either way, intubation or general anesthesia for SPEN alone was not necessary.

**Fig. 3:**
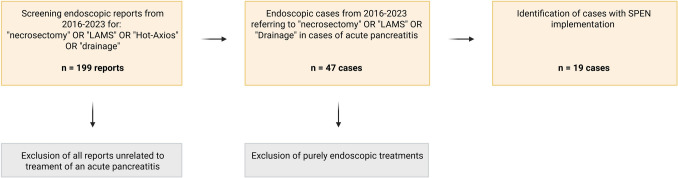
Flowchart of patient selection

The retrospective and descriptive analysis was conducted in accordance with the approval of the local ethics review board (EK030/19). Patient selection was performed as follows: an initial screening of endoscopy reports containing the keywords “necrosectomy,” “LAMS,” “Drainage,” or “Hot-Axios” between 2016 and 2023 identified 199 endoscopic reports. After exclusion of all reports unrelated to the management of an acute pancreatitis, 47 cases involving a necrosectomy were retained. Subsequently, purely transgastric endoscopic procedures were excluded, resulting in a final cohort of 19 cases for further analysis. A flowchart illustrating the patient selection process is shown in Fig. 3. Descriptive analysis was included patient’s characterization, examination of disease severity as well as evaluation of therapy regime and procedure-associated complications. A detailed summary of the complications screened is stated in Table 1. Results were compared with current data acquired by literature research.

**Table 1:** Disease complications

*Complications*	*Definition*	*Comment*
***Disease-related organ failure***		
New-onset single or multiple organ failure or systemic complications	New-onset failure (i.e., not present at any time before first intervention) of one in case for single or two or more organs for multiple organ failure. Alternatively, occurrence of two or more systemic complications at the same time	–
(Acute) Pulmonary failure	Respiratory insufficiency with need for mechanical ventilation	Further detailed definition analogous to the Berlin Definition of ARDS from 2012 [1]
(Acute) Circulatory failure/Sepsis	Circulatory systolic blood pressure < 90 mmHg despite adequate fluid resuscitation or need for inotropic support Septic shock is defined as persistent hypotension despite adequate fluid substitution with a subsequent need of vasopressor therapy in order to maintain a mean arterial blood pressure of ≥ 65 mmHg. Simultaneously lactate value must exceed > 2 mmol/l	German Sepsis Guideline [2, 3]
(Acute) Renal failure	Rise of serum creatinine ≥ 0,3 mg/dl (26,5 μmol/l) or elevation greater 1,5- bis 1,ninefold of the initial value	German Guideline for Renal Failure [4]
***Disease-related systemic complication***		
DIC	Platelet count < 150 × 10^9^ Liter	-
Abdominal compartment syndrome	Intra-abdominal pressure > 20 mmHg requiring surgical treatment	Guideline from the World Society of the Abdominal Compartment Syndrome [5]
Endocrine pancreatic dysfunction	New-onset needs for drugs in order to sustain glucose homeostasis	–
Exocrine pancreatic dysfunction	New-onset needs for oral pancreatic-enzyme supplementation	–
***Intervention-associated complication***		
VARD-associated complications	Complication with immediate temporal association (< 30 days) with the procedure	Severity grading according to Clavien–Dindo Classification [6]
SPEN-associated complications		

## Results

### Patient characteristics

A total of 19 patients (7 male, 12 female) with an average age of 54.9 ± 13.0 years and a mean BMI of 29.5 ± 8.2 kg/m^2^ were included in this study (Table 2 depicts the patient characteristics).

**Table 2:** Patient characteristics

**Characteristics**	
Age (years)	54.9 ± 13.0 (mean ± SD)
Female sex	*n* = 12
Male sex	*n* = 7
BMI (kg/m^2^)	29.5 ± 8.2 (mean ± SD)
Etiology (incidence)	
Biliary (gallstone)	*n* = 8 (42.1%)
ERCP-related	*n* = 4 (21.1%)
Alcohol-related	*n* = 5 (26.3%)
Idiopathic	*n* = 2 (10.5%)
Infection rate (incidence)	
Confirmed local infection*	*n* = 19 (100%)
Confirmed systemic infection**	*n* = 9 (47%)
**Comorbidities and risk assessment**	
Coexisting comorbidities (incidence)	
Diabetes mellitus	*n* = 4 (21.1%)
CKD	*n* = 1 (5.3%)
Coronary atherosclerosis	*n* = 1 (5.3%)
COPD	*n* = 0 (0%)
ASA score (incidence)	
< III	*n* = 0 (0%)
III	*n* = 14 (73.7%)
IV	*n* = 3 (15.8%)
V	*n* = 2 (10.5%)

^*^Positive pathogen detection in peripancreatic tissue samples

^**^ Positive blood culture testing

The most common cause of acute pancreatitis was biliary in origin, accounting for 42.1% of cases, primarily due to gallstone obstruction leading to pancreatic fluid stasis. The second most frequent cause was alcohol-related pancreatitis, observed in 26.3% of patients. Nearly the same proportion of patients (21.1%) developed pancreatitis following endoscopic retrograde cholangiopancreatography (ERCP). In 10.5% of cases, the underlying cause remained unidentified and was classified as idiopathic.

In all patients, bacterial pathogens could be isolated from necrotic tissue samples obtained through a percutaneous approach, confirming pancreatic necrosis superinfection. However, only 47.4% of patients tested positive for bacteremia.

To assess frailty and comorbid conditions, the determination of the American Society of Anesthesiologists (ASA) classification interestingly indicated a high frailty rate in the patient collective, despite a relatively low prevalence of preexisting conditions (Table 2). All patients had an ASA score of at least III, with 14 patients classified as ASA III, 3 as ASA IV, and 2 as ASA V.

### Disease severity

Actual disease severity was assessed by examination of the occurrence of organ failure and systemic complications (Table 3). The study cohort predominantly consisted of critically ill patients, as the majority of 78,9% developed new-onset organ dysfunction associated with their primary condition (Table 3), with sepsis occurring in 57.9% of cases, acute kidney injury (AKI) in 52.6%, and acute respiratory distress syndrome (ARDS) in 26.3%. Additionally, 21.2% of patients experienced recurrent septic episodes (Table 3). In sum, 57.9% of patients even developed new-onset multiple organ dysfunction (MOD) in terms of dysfunction of at least two organ systems. MOD included different combinations of organ failure.

**Table 3:** Major complications associated with the primary disease

Patients w/o organ dysfunction (incidence)	21.1%
Patients with organ dysfunction (incidence)	*n* = 15 (80%)
Single organ dysfunction	*n* = 4 (21.1%)
Multiple organ dysfunction (MOD)	*n* = 11 (57.9%)
Sepsis	*n* = 4 (57.9%)
Recurrent septic episodes	*n* = 4 (21.1%)
Acute kidney injury (AKI)	*n* = 10 (52.6%)
ARDS	*n* = 5 (26.3%)
Other systemic complications (incidence)	
Abdominal compartment syndrome	*n* = 3 (15.8%)
Endocrine pancreatic insufficiency	*n* = 1 (5.3%)
Exocrine pancreatic insufficiency	*n* = 2 (10.5%)
Thrombocytopenia	*n* = 2 (10.5%)
Mortality (incidence)	*n* = 3 (15.8%)
30-day mortality	*n* = 1 (5.3%)

Other major systemic complications related to the disease included abdominal compartment syndrome in 15.8% of patients, necessitating surgical decompression via laparotomy. New-onset exocrine pancreatic insufficiency was observed in 10.5% of cases, while another 10.5% developed disseminated intravascular coagulation (DIC) with thrombocytopenia (Table 3). New-onset endocrine pancreatic insufficiency was documented in one patient.

This high incidence in major disease-related complications eventuated not only in a prolonged hospitalization interval of 108.32 ± 54.44 days on average but also on a high percentage of 78.9% of patients presented with an indication for ICU admission at any point of their treatment history. Among all patients who were in need for ICU treatment, the large majority of 93.3% had their first ICU admission prior to any intervention and only one patient had their first ICU admission after the first intervention. Almost half of the patients required a re-admission to the ICU department after temporary transfer to the regular ward, resulting in a mean ICU admission rate of 1.6 ± 1.3 with a range of 1 to 5 per individual (Table 4). Cumulative ICU stay was 44.9 ± 57.5 days on average. Despite ICU care, 15.8% of patients succumbed to their condition, resulting in three deaths within this cohort. The 30-day mortality rate was recorded at 5.3% (Table 3).

**Table 4:** Hospitalization statistics

Overall hospital stay	108. ± 54.4 days (mean ± SD)
ICU	
Admissions total (incidence)	*n* = 15 (78.9%)
Re-admissions (incidence)	*n* = 9 (47.4%)
Admissions per patient	1.6 ± 1.3 (mean ± SD, range = 1—5)
Initial admissions prior to intervention (incidence)	*n* = 14 (93.3%)
Initial admissions after to intervention (incidence)	*n* = 1 (6.7%)
Cumulative stay	44.9 ± 57.6 days (mean ± SD)

### Treatment regime

Regarding initial drainage treatment, a minority of five patients (31.6%) primarily received an EUS-guided transgastric drainage prior to a CT-guided percutaneous drainage. Transgastric drainage was realized by implantation of either a self-expanding metal stent (SEMS, 10.5%) or a Lumen-Apposing Metal Stent (LAMS, 15.8%) with or without a transgastric pigtail drainage (15,8%). In all patients, transgastric drainage showed insufficient evacuation of the existing necrosis, eventually, leading to the placement of a CT-guided percutaneous drainage. Thus, all patients received a CT-guided percutaneous drainage prior to VARD (Table 5).

**Table 5:** Procedure statistics

EUS-guided drainage (incidence)	*n* = 6 (31.6%)
LAMS	*n* = 3 (15.8%)
SEMS	*n* = 2 (10.5%)
Transgastric pigtail drainage through a LAMS	*n* = 3 (15.8%)
CT-guided drainage (incidence)	*n* = 19 (100%)
VARD (incidence)	*n* = 19 (100%)
Repetitive VARD	*n* = 6 (31.6%)
SPEN (incidence)	*n* = 19 (100%)
Repetitive SPEN (incidence)	*n* = 15 (78.9%)
# of SPEN applications	4.3 ± 3.8 (mean ± SD, range = 1—14)
Interval between SPEN treatments (days)	6.9 ± 3.3 (mean ± SD, range = 3—11)
SPEN treatment period (days)	20.9 ± 24.7 (mean ± SD, range = 0 – 84)

As all patients exhibited therapy-refractory disease, all patients subsequently underwent surgical necrosectomy via VARD. Among all patients, 31.6% required repeated VARD procedures due to extensive necrosis formation, necessitating further necrosectomy (Table 5).

**Fig. 4:**
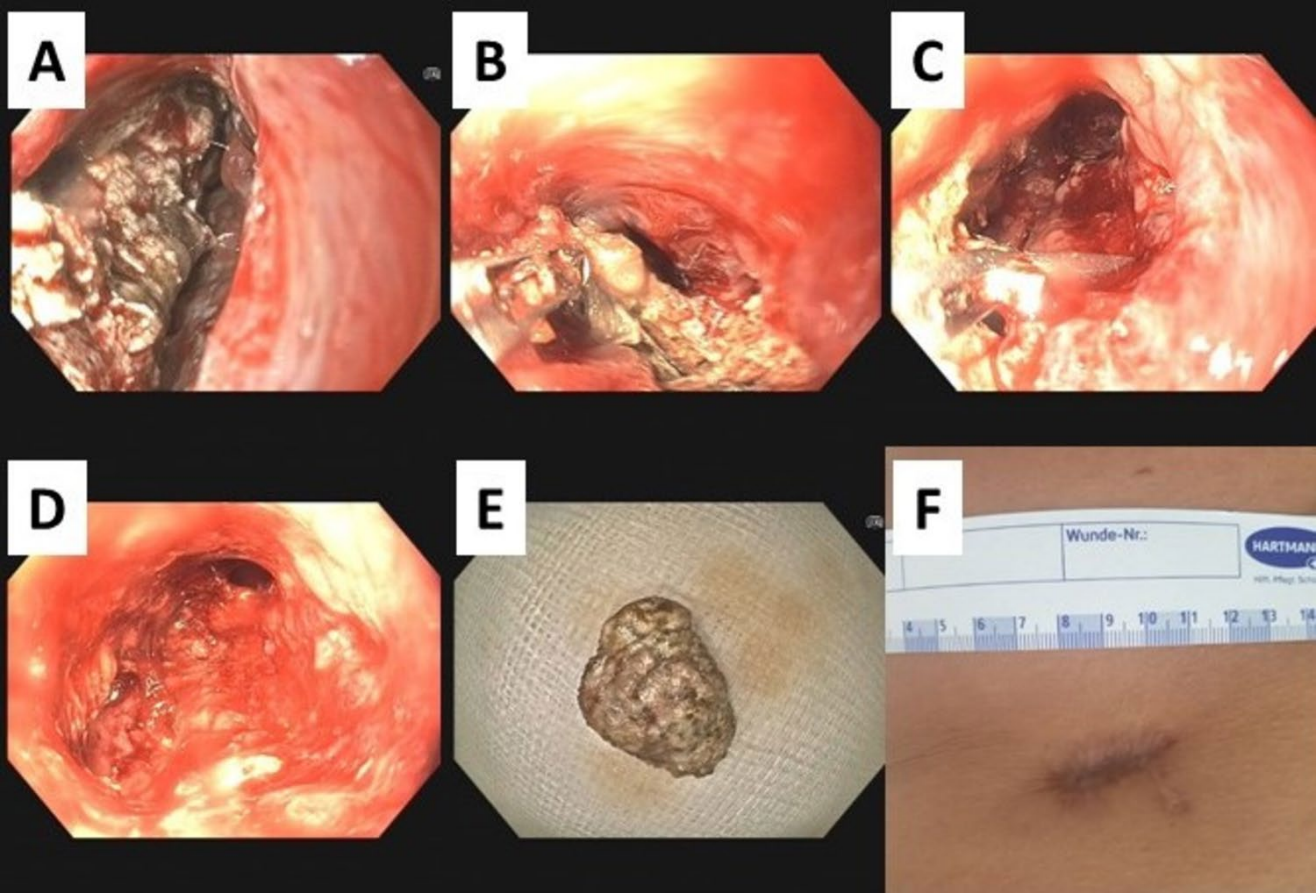
SPEN procedure

Alike drainage treatment, additional VARD treatment alone remained unsuccessful to sustainably clear all necrosis formation. Aiming to provide a less traumatic alternative to VARD, ultimately, SPEN was established. SPEN was performed on all patients with varying frequencies corresponding to the individual needs. While the minority of patients experienced adequate necrosis elimination with no further need for intervention upon singular SPEN treatment, patients received 4.3 ± 3.8 applications (range = 1 – 14) on average over the course of 20,9 ± 24,7 days (mean ± SD, range = 0 – 84 days). The interval between SPEN sessions ranged from 3 to 11 days (mean 6.9 ± 3.3 days), corresponding to an average weekly repetition when indicated. Figure 4 presents a typical view presented to the physician performing SPEN. Faced necrosis formations (Fig. 4A) could be removed using endoscopic devices (Figs. 4B–D, 2F) until the necrosis was successfully evacuated (Fig. 4E). Figure 4G shows the residual minor scar after successful treatment with SPEN.

### Procedure-related complications

Procedure-related complications were retrospectively examined and classified using the Clavien–Dindo system, distinguishing between minor complications (Clavien–Dindo Grade II) and significant complications (Clavien–Dindo ≥ Grade III) ^21^. In general, VARD was associated with higher complication rates compared to SPEN (31,6% vs. 10,5%). Although the incidence of minor complications (Clavien–Dindo Grade II) was identical for both procedures, VARD complications were generally more severe. Notably, while SPEN resulted in no significant complications (Clavien–Dindo ≥ Grade III), VARD accounted for four significant complications (21%) (Table 6). Based on the observed complication rates, we concluded that SPEN represents a less traumatic alternative to Re-VARD, and, therefore preferred SPEN over Re-VARD.

**Table 6:** Major procedure-associated complications*

VARD-associated complications according to Clavien–Dindo classification (incidence)	*n* = 6 (31.6%)
Clavien–Dindo °II**	*n* = 2 (10.5%)
Clavien–Dindo °IIIb***	*n* = 2 (10.5%)
Clavien–Dindo °IVb****	*n* = 2 (10.5%)
SPEN-associated complications according to Clavien–Dindo classification (incidence)	*n* = 2 (10.5%)
Clavien–Dindo °II*****	*n* = 2 (10.5%)

^*^Occurrence < 30 days after the procedure und directly associated with the procedure rather than the primary disease

^**^ Induction or Escalation of antibiotic therapy (*n* = 2)

^***^ Enterocutaneous Fistula (*n* = 1), Bleeding (*n* = 1)

^****^ Dialysis (*n* = 2), ARDS with need of inhalative NO treatment (*n* = 1)

^*****^ Induction antibiotic therapy (*n* = 1), Venous bleeding (ceased spontaneously, *n* = 1)

## Discussion

We report on a therapeutic approach combining two minimally invasive treatments: VARD and subsequent SPEN. The combination of these two methods showed promising therapeutic success by leveraging the most effective features of each treatment into a synergistic therapy regimen. While VARD enabled extensive surgical necrosectomy, SPEN provided a complementary, effective, yet less traumatic dynamic necrosectomy. Additionally, SPEN helped conserve both, human and material resources, as it did not require treatment in a surgical theater. This was possible because SPEN could be performed under analgosedation without the explicit need for general anesthesia or an operating room team.

When integrating our findings into current clinical practice and existing clinical data, the investigated patient population aligns with the statistical characteristics of acute pancreatitis (AP) cases, as the majority were associated with either biliary or alcohol-related etiology [3]. Similarly, in this specific group of severely sick patients both the complication and mortality rates matched previously reported statistical measures [3]. The mortality rate of 15.8% was in coherence with the statistical death rates associated with necrotizing pancreatitis [6], although in recent years there appears to be a decrease in mortality rates [1]. Given that we aimed to explore a novel treatment approach for patients with therapy-refractory disease, the investigated patient cohort primarily consisted of severe NP cases.

Regarding procedure-related complications, VARD was associated with two minor complications, specifically reactive fever episodes, likely due to the lancing of infected necrotic areas, which exposed a larger surface to potential pathogens and triggered a systemic inflammatory response. In both cases, adequate control was achieved through the initiation or escalation of antibiotic therapy. Among major complications, we observed one enterocutaneous fistula and one active venous bleeding. The most severe complications occurring in close temporal association with VARD were acute kidney injury requiring dialysis and acute respiratory distress syndrome (ARDS) necessitating inhaled NO therapy (Table 3). Nonetheless, VARD is regarded as an essential component of this treatment approach, as it allows for extensive necrosectomy when required.

In contrast, SPEN demonstrated beneficial features as it was associated with lower complication rates and less severe adverse events compared to VARD (Table 6). Despite the lack of large-scale randomized controlled trials on this technique, our findings are in line with existing data demonstrating the safety and feasibility of percutaneous necrosectomy at the bedside [13, 22]. Beyond the inherently less traumatic nature of the procedure itself, the avoidance of anesthesia-related risks may further contribute to the beneficial safety profile of SPEN. Considering the previously reported comparable resolution rate for PEN (~ 82%) [22] and VARD (50–83%) [23], we suggest that SPEN may present the technique of choice for patients with therapy-refractory disease after initial VARD, owing to its advantageous safety and flexibility profile at comparable therapeutic success rates. Particularly the flexibility of this approach enables valuable implications for patients in need of ICU treatment as ICU-bound patients may benefit from bedside treatment under light analgosedation rather than transport into an operating theater and surgery under general anesthesia. Or, in other words, such patients may benefit from this concept of “bringing the treatment to the patients,” rather than “bringing the patient to the treatment.”

Overall, SPEN via the VARD access showed promising potential as a further step toward minimally invasive treatment of therapy-refractory infected necrotizing pancreatitis. We believe that the greatest benefit arises from the combination of an initial extensive necrosectomy via VARD and the convenience of the less traumatic SPEN procedure, which can be adapted flexibly to meet individual patient needs.

## Limitations

First and foremost this study presents with the limitation of a presenting a retrospective single-center study investigating a small patient cohort. Furthermore, considering SPEN to be a novel treatment approach with no data guiding the decision between introduction of Re-VARD and immediate SPEN administration, Re-VARD was still performed in 31,6% of all cases (Table 5). Moreover, this technique could be compared to alternative therapeutic approaches, such as single-port robotic necrosectomy via the VARD channel. Considering the cohort size, the demonstrated protocol may be regarded as an investigational approach. Future prospective studies must be conducted to, firstly, establish a reliable SPEN protocol, and, secondly, provide a controlled comparison between SPEN and existing procedures. Likewise, additional randomized prospective trials could be conducted to establish reliable criteria to distinguish between indications for either SPEN or Re-VARD.
